# Are anxiety and depression associated with cognition and cardiovascular function in young male and female adults?

**DOI:** 10.1371/journal.pone.0292246

**Published:** 2023-10-18

**Authors:** Florine Ruthmann, Nadia Guerouaou, Francis Vasseur, Maria-Claire Migaud, Dominique Deplanque, Frederic Gottrand, Laurent Beghin, Odile Viltart

**Affiliations:** 1 PsySEF Département, Univ. Lille, SCALab—Sciences Cognitives et Sciences Affectives, UMR CNRS 9193, Lille, France; 2 Univ. Lille, INSERM, CHU Lille, UMR-S 1172, LilNCog Lille Neuroscience & Cognition, Degenerative and Vascular Cognitive Disorders, Lille, France; 3 Univ. Lille, INSERM, CHU Lille, CIC1403—Centre d’Investigation Clinique, Lille, France; 4 EA 2694—Département de Biostatistiques, Univ. Lille, CHU Lille, Lille, France; 5 Univ. Lille, INSERM, CHU Lille, U1286—INFINITE—Institute for Translational Research in Inflammation, Lille, France; 6 Univ. Paris, Institut de Psychiatrie et Neuroscience de Paris (IPNP), INSERM, U1266, Vulnerability of Psychiatric and Addictive Disorders, Paris, France; McMaster University, CANADA

## Abstract

The results of recent studies suggested that emotional disorders (such as anxiety and depression), cognitive impairments and cardiovascular disorders are related on the subclinical level. These major health issues are often concomitant and have complex, sex-dependent relationships; it is therefore important to study these issues concomitantly in the general population, in order to gain a better understanding of early-stage subclinical relationships between these conditions. The objective of this exploratory study was to assess correlations between anxiety, depression, cognition, and endothelial function in young adults from the general population. Endothelial function (*via* the reactive hyperaemia index (RHI) was assessed with a plethysmographic device. Depression and anxiety were self-reported *via* the Beck Disorder Inventory II and the State-Trait Anxiety Inventory, respectively. The Cambridge Neuropsychological Test Automated Battery was used to measure performances in visuospatial memory, visuospatial working memory, and sustained attention. Performances in inhibition and flexibility were evaluated with the Color Word Interference Test. Forty-four young adults (21 males; mean ± standard deviation age: 25.8 ± 1.1; 23 females; mean age: 25.6 ± 1.4) were included in the study. Anxiety was correlated with a low RHI (*r* = -0.40, p = 0.015, 95% CI [-0.64, -0.08]). In females, the depression score was positively correlated with the number of errors in the visuospatial memory task (*r* = 0.42, p = 0.049; 95% CI [-0.002, 0.70]) and visuospatial working memory (*r* = 0.57, p = 0.005; 95% CI [0.10, 0.79]). In males, high anxiety and depression scores were negatively correlated with the number of errors in visuospatial working memory task (anxiety: *r* = -0.77, p = 0.001; 95% CI [-0.91, -0.43]; depression *r* = -0.61, p = 0.004, 95% CI [-0.82, -0.22], respectively). However, the relationship between cognitive performance and RHI was not significant. Our data suggest that anxiety and depression could be differentially related to cognitive and endothelial functions in a non-clinical population of young adults. More research is needed to confirm these results, understand the pathophysiological mechanisms in more details, and assess the importance of a sex-specific approach.

## Introduction

Anxiety and depression are currently the most common emotional disorders and are more frequent among females than males [1]. These disorders are associated with deleterious cognitive and physiological alterations [2]. Longitudinal studies demonstrated that patients suffering from emotional disorders have a greater risk of developing both cognitive impairment and cardiovascular disease at old age [3–5]. The nature of cognitive impairments in emotional disorders has been largely studied without a clear-cut consensus [6–9]. Recently, cardiovascular function has been suggested to mediate such cognitive impairments in emotional disorders [10].

Prospective studies showed that individuals experiencing anxious and depressive disorders have a greater risk of developing cardiovascular diseases (CVD), independently of traditional cardiovascular risk factors [10–12]. Indeed, altered endothelial function, a reliable biomarker to assess cardiovascular function, was associated with anxiety traits among old adults but not in young male population [13]. Moreover, self-reported anxiety and depression were positively correlated with endothelial function in female adolescents, but not male adolescents, from the general population [14]. The relationship between cognitive impairment and cardiovascular function has also been extensively investigated, as longitudinal studies established that older adult with CVD are at greater risk of cognitive decline or dementia [15]. More recently, these results were found to middle-aged adults, in whom better cardiovascular function is linked to better cognitive functions [16–18], raising the question of the times at which the various entities appear. There is currently a body of evidence for relationships between anxiety/depression, cognitive impairments, and cardiovascular dysfunction. However, these three factors are rarely studied simultaneously. In consequence, their inter-relationships are not clearly defined. This problematic also requires further investigation in young adults, a population in which prevention is likely to be more effective. The potential sex-specificity of these relationships must also be considered carefully.

In view of the previous studies described above, we hypothesized correlations between high levels of anxiety and depression, low cognitive scores, and poor endothelial function. We also hypothesize that these correlations are sex-dependent. To validate such assumptions, our objective was therefore to investigate the associations between anxiety/depression, cognitive functions, and endothelial functions in male and female young adults from the general population.

## Material & methods

### Population and study design

This was an ancillary study of the Better Life by Nutrition During Adulthood (BELINDA) population (ClinicalTrials.gov: NCT02899416). The study was approved by the local independent ethics committee (*CPP 19/29 Nord Ouest IV*, Lille, France; reference: 2016 A00386 45). The objectives of the BELINDA study were to evaluate cardiovascular risks (using the Pathobiological Determinants of Atherosclerosis in Youth risk score) during young adulthood (age: 21–32) and determine risk factors (including neuropsychological factors) during adolescence [19].

In the present ancillary study, all the participants have been attending the BELINDA investigating centre at Lille University Hospital (Lille, France).

The participants were all part of the initial Healthy Lifestyle in Europe by Nutrition in Adolescence (HELENA) study, which was designed to investigate cardiovascular risk factors in adolescents [20]. Other main criteria for inclusion in the BELINDA study were social security coverage and written informed consent. Criteria for non-inclusion were pregnancy, breastfeeding, incarceration, and inability to provide informed consent. Among the participants in the BELINDA study, we have included in the analyses the 44 participants for whom a measure of endothelial function was available.

After day admission to hospital, endothelial function has been measured at 9:00 am. At 11:00 am, participants have completed a neuropsychological assessment that included self-questionnaires (on anxiety and depression) and a cognitive evaluation.

### Measurement of endothelial function

Endothelial function has been measured using the EndoPAT® 2000 plethysmographic device (Itamar Medical, Caesarea, Israel). This non-invasive device measures the change in digital blood volume induced by transient forearm ischemia; the technique is also known as peripheral pulse tonometry. The results are strong predictors of major cardiac events [21].

Endothelial reactivity was analysed using digital probes with an inflatable internal membrane system connected to the recording device. Plethysmographic probes were placed on the index finger of each hand, in order to measure changes in pulse wave amplitude (PWA, reflecting changes in blood volume). The baseline PWA was recorded continuously for 15 minutes, while the participant was lying down. The uninflated pressure cuff was placed around the forearm. The contralateral arm served as a control. The PWA was then measured for a further period of 5 minutes. Hyperaemia was initiated by occlusion of the brachial artery blood flow for 5 minutes, using a pressure of 50 mmHg more than the systolic blood pressure. The PWA was recorded for 5 minutes after the pressure cuff had been released and thus yielded a measure of post-ischemic digital hyperaemia. The reactive hyperaemia index (RHI) was calculated automatically by the software linked to the EndoPAT® 2000 device (Itamar Medical) as the ratio between the mean PWA during the occlusion (from 90 to 120 seconds) and the mean PWA for the 210 seconds prior to the occlusion. To eliminate the confounding systemic neurovegetative vasomotor effect, the RHI was normalized against the contralateral arm. A low RHI indicates poor endothelial function and thus a high cardiovascular risk.

### Self-assessment of emotional disorders

The participants have filled out two validated and widespread used self-report inventories to assess anxiety and depression, respectively. The State Trait Anxiety Inventory (STAI) consists of two different 20-item questionnaires (including reverse item) for both state anxiety (STAI-S) and trait anxiety (STAI-T) [22]. These questionnaires are based on a four-point scale from “almost never” to “almost always”. Scores range from 20 to 80, with higher score indicating greater anxiety. In the present study, a STAI score of ≥46 corresponds to at least a moderate level of anxiety.

The Beck Depression Inventory II (BDI-II) consists of 21 items to assess the presence and severity of depressive syndromes [23]. Each item comprises four possible statements and is scored from 0 to 3. Final scores range from 0 to 63, with higher score indicating more severe depression. Here, a score of ≥14 corresponds to a significant level of depression.

### Cognitive assessments

The participants first have completed the Cambridge Neuropsychological Test Automated Battery a validated, computerized battery of neurocognitive tests that has been used to measure sustained attention, visuospatial memory, and visuospatial working memory [24]. Data have been collected automatically by the CANTAB software. Participants used a touch-sensitive tablet and a joystick with two buttons. The Rapid Visual Information Processing (RVP) test measured sustained attention, the Paired Associates Learning (PAL) test measured visuospatial memory, and the Spatial Working Memory (SWM) test measured visuospatial working memory. Then, the participants have completed the Delis-Kaplan Executive Function System Color Word Interference Test (CWIT) test, which uses the Stroop effect to measure inhibitory control and cognitive flexibility [25]. The CWIT consists of four reading boards and colour naming under simple, inhibition and flexibility conditions.

### Statistical analyses

Continuous variables are reported as the mean ± standard deviation or the median [interquartile range (IQR)] for the whole sample and for males and females separately.

The participant’s sex was transformed into a binary numerical variable: 0 for males and 1 for females. In order to select the appropriate statistical test (parametric or non-parametric), we assessed normality and equality of variances with the Shapiro-Wilk (S-W) test. We next applied a Conover and Iman rank transformation procedure so that standard parametric tests could be applied to the data’s rank rather than the data *per se* [26].

We used Student’s t-test to probe for differences between males and females and calculated effect size with the Cohen’s d, with higher values indicating larger magnitude of the effect. The Levene variance homogeneity test was applied to all variables.

In order to investigate the relations between anxiety, depression, endothelial function and cognition, correlations were performed with the Bravais-Pearson linear correlation analyses. The correlation coefficient *r* and the p value were calculated for each pair of variables for the sample as a whole and for each sex separately.

To determine whether endothelial function, anxiety and depression influenced the cognitive scores, we performed five linear regressions (one per cognitive score) in the whole sample with a maximum 10 participants per independent variable [27], the cognitive scores as dependant variables, and the STAI-T score, BDI-II score, and RHI as independent variables. These multiple linear regressions were also performed separately in males and females. It is important to note that, in this case, there were only 5 to 10 participants per independent variable; this reduced the accuracy and reliability of the results. The threshold for statistical significance was set to p<0.05. The statistical analyses were performed with Statistica Data Science Workbench software (version 14, TIBCO Software Inc., Palo Alto, CA, USA).

## Results

### Study population and anxiety/depression results

Our analysis covered 44 participants (mean age: 25.7 ± 1.3), with 21 males (mean age: 25.8 ± 1.1) and 23 females (mean age: 25.6 ± 1.4). Only the data on trait anxiety were normally distributed (S-W = .983, p = .835) (Table 1).

**Table 1:** Mean scores, standard deviations, medians, interquartile ranges, and the number of available data for variables measuring the cardiovascular risk (RHI), emotional disorders (BDI-II, STAI State, and STAI Trait), and cognition (CWIT inhibition and flexibility, PAL errors, RVP hit, and SWM errors). n: number of subjects included in this study.

		RHI	BDI-II	STAI-S	STAI-T	CWIT inhibition score	CWIT flexibility score	PAL errors	RVP Hit	SWM errors
**Pooled sample**	Mean	1.99	7.34	29.17	39.05	10.81	9.29	10.98	0.64	21.74
	*SD*	(0.6)	(5.6)	(6.4)	(8.3)	(2.5)	(2.7)	(10)	(0.2)	(17.9)
	Median	1.9	6.5	28.5	38	11	9	8.5	0.65	17
	IQR	0.54	8	9.25	10.75	2	3	10.75	0.25	16.75
	n =	42	44	40	38	44	44	42	42	42
**Females**	Mean	1.98	8.91	30.36	42.09	10.43	9.65	12.7	0.58	20.36
	*SD*	(0.6)	(5.2)	(6.4)	(7.1)	(2.9)	(3.2)	(10.6)	(0.2)	(17.3)
	Median	1.87	8	29	42	11	10	9.5	0.6	16.5
	IQR	0.49	6	8.5	8.75	2	2.5	11.5	0.25	19.5
	n =	21	23	22	22	23	23	22	22	22
**Males**	Mean	2.00	5.62	27.72	34.87	11.23	8.90	9.00	0.72	23.25
	*SD*	(0.6)	(5.5)	(6.3)	(8.3)	(2)	(2)	(9)	(0.04)	(18.8)
	Median	1.9	3	26.5	35	11	9	8.5	0.71	21.5
	IQR	0.48	6	10.5	10.75	2	2	9	2.29	16.25
	n =	21	21	18	16	21	21	20	20	20

BDI-II: Beck Depression Inventory II; CWIT: Color Word Interference Test; IQR; interquartile range; PAL: Paired Associated Learning; SD: standard deviation; STAI-S: State Trait Anxiety Inventory, State Anxiety; STAI-T: State Trait Anxiety Inventory, Trait Anxiety; SWM: spatial working memory; RHI: reactive hyperaemia index; RVP: rapid visual information processing

According to the questionnaire scores, 13.6% (three males and three females) of the participants had a significant level of depression, 23.7% of the participants (seven females and two males) had at least a moderate level of trait anxiety, and none of the participants had state anxiety. 10.5% of participants who completed the questionnaires (three females, one male) had significant levels of both depression and trait anxiety.

### Sex differences

A one-tailed t-test revealed that BDI-II scores were higher in females (mean: 8.9 ± 5.2) than in males (5.6 ± 5.5; *t* = -2.43, *p* = 0.01, *d* = - 0.73) and also that STAI-T scores were higher in females (mean: 42.1 ± 7.1) than in males (34.9 ± 8.3), (*t* = -2.77, *p* = 0.004, *d* = - 0.909). A two-tailed t-test showed that the probability of hits in the RVP test was higher in males (mean: 0.73 ± 0.04) than in females (0.58 ± 0.2), (*t* = 2.42, *p* = 0.02, *d* = 0.75). No other significant differences were found.

### Correlations between anxiety, depression, cognitive scores, and cardiovascular scores

STAI-S and STAI-T were positively correlated in the study population as a whole (*r* = 0.54, p = .001; 95% CI [0.24, 0.74]) (Table 2). The BDI-II score was also positively correlated with both STAI-S (*r* = 0.41, p = 0.009, 95% CI [0.11, 0.63]) and STAI-T (*r* = 0.60, p < 0.001, 95%CI [0.34, 0.77]). In line with our hypotheses, RHI was negatively correlated with both STAI-S (*r* = -0.40, p = 0.012, 95% CI [-0.63, -0.9]) and STAI-T (*r* = -0.40, p = 0.015, 95% CI [-0.64, -0.08]). Lastly, a high BDI-II score was negatively correlated with the probability of hits in the RVP test (*r* = -0.31, P = 0.046, 95% CI [-0.56, -0.007]). No other correlations were found.

**Table 2:** Correlation matrix for the variables measuring emotional disorders (BDI-II, STAI-S, STAI-T), the cardiovascular risk (RHI), and cognition (CWIT-I, CWIT-F, PAL-e, RVP-hit, and SWM-e) in the pooled sample (r and p values).

	RHI	CWIT-I	CWIT-F	PAL-e	RVP-hit	SWM-e
RHI	--	-0.15	0.13	0.10	-0.22	0.10
BDI-II	-0.18	-0.16	0.06	-0.04	**-0.31** *	0.18
STAI-S	**-0.40** *	-0.12	0.28	-0.13	-0.09	-0.4
STAI-T	**-0.40** **	-0.22	0.16	-0.19	-0.06	0.07

* p < .05

** p < .01

BDI-II: Beck Depression Inventory II; CWIT: Color Word Interference Test; PAL: Paired Associated Learning; STAI-S: State Trait Anxiety Inventory, State Anxiety; STAI-T: State Trait Anxiety Inventory, Trait Anxiety; SWM: Spatial Working Memory; RHI: Reactive Hyperaemia Index; RVP: Rapid Visual Information Processing

Interestingly, males and females had different correlation patterns (Table 3).

**Table 3:** Correlation matrix for the variables measuring emotional disorders (BDI-II, STAI-S, STAI-T), the cardiovascular risk (RHI), and cognition (CWIT-I, CWIT-F, PAL-e, RVP-hit, and SWM-e) in the pooled sample (r and p values).

	RHI		CWIT-I		CWIT-F		PAL-e		RVP-hit		SWM-e	
	M	F	M	F	M	F	M	F	M	F	M	F
RHI	-	-	-0.005	-0.3	0.04	0.23	0.24	0.004	-0.3	-0.21	0.24	-0.03
BDI-II	-0.02	-0.38	-0.17	-0.08	-0.08	0.06	**-0.61** **	**0.42** *	-0.12	-0.31	-0.09	**0.57** **
STAI-S	-0.28	**-0.54** *	-0.21	-0.02	0.13	0.32	-0.41	0.01	-0.33	0.28	-0.13	0.09
STAI-T	**-0.50** *	-0.27	-0.46	-0.02	0.33	-0.07	-0.77***	0.15	0.23	-0.05	0.13	0.12

* p < .05

** p < .01

BDI-II: Beck Depression Inventory II; CWIT: Color Word Interference Test; PAL: Paired Associated Learning; STAI-S: State Trait Anxiety Inventory, State Anxiety; STAI-T: State Trait Anxiety Inventory, Trait Anxiety; SWM: Spatial Working Memory; RHI: Reactive Hyperaemia Index; RVP: Rapid Visual Information Processing

In females, RHI was negatively correlated with STAI-S (*r* = -0,54, p < 0.05, 95% CI [-0.8, -0.12]) but not correlated with STAI-T. BDI-II was positively correlated with the number of errors in the PAL test (*r* = 0.42, p = 0.049; 95% CI [-0.002, 0.70]) and the SWM test (*r* = 0.57, p = 0.005; 95%CI = [0.10, 0.79]). No other correlations were found regarding our hypotheses.

In males, STAI-T was negatively correlated with RHI (*r* = -0.50, p = 0.05; 95%CI = -[0.78, -0.006]). In contrast to the results obtained with females, the number of errors in the PAL test was negatively correlated with BDI-II (*r* = -0.61, p = 0.004, 95% CI [-0.82, -0.22]) and STAI-T (*r* = -0.77, p = 0.001; 95% CI [-0.91, -0.43]). No other correlations were found regarding our hypotheses.

### Prediction of cognitive scores

In the total sample, no multiple linear regression was significant (data not shown).

In females, the multiple linear regression predicting the number of SWM errors was significant (R^2^ = 0.50, adjusted R^2^ = 0.41, F = 5.1, p = 0.012). It was found that BDI-II score significantly predicted the number of SWM errors. (β = 0.92, p = 0.002)

In males, the multiple linear regression predicting the number of PAL errors was significant (R^2^ = 0.71, R^2^ adjusted = 0.63, F = 9,61, p = 0.002). It was found that STAI-Trait significantly predicted the number of PAL errors (β = -0.97, p = 0.003).

## Discussion

The objective of the present exploratory study was to understand the relationships between anxiety, depression, cognition, and endothelial function in young adults. Our results highlight a correlation between self-reported anxiety and depression levels and cognitive performances and a correlation between high self-reported levels of anxiety and a low RHI.

### Anxiety and depression are both correlated with cognitive function but in different ways

The relationships between emotional disorders and cognition have been the subject of much research, from Beck’s early cognitive theory to more recent neuroimaging studies [28, 29]. In the present study, although anxiety and depression were both associated with cognitive performances, the correlations differed and were sex-dependant. Firstly, and in contrast to our starting hypothesis, our results did not suggest that a high level of anxiety is correlated with poor cognitive performance. In the literature, impairments in cognitive performance appear to depend on the subtype of anxiety [9] this factor was not considered in our study. For example, a recent study found that cognitive performances are maintained in individuals with generalized anxiety disorders [30]. This is consistent with the attentional control theory, in which cognitive performances can be maintained if the individual implements compensatory strategies (such as an increased cognitive effort) [31]. Thus, the putative relationship between trait anxiety and cognitive performance in our young adult population might have been absent or not strong enough to be detected.

However, in males, we observed an unexpected correlation between high trait anxiety and good spatial memory performances. This might be related to sex differences in visuospatial memory [32]. It is also possible that the most anxious participants compensated by increasing their cognitive effort [33, 34]. Finally, mild anxiety could have different effects on neuropsychological testing performances (decline, improvement, or no effect) depending on the sex of the participants [34].

In accordance with our starting hypotheses, we found that depression was correlated with sustained attention, spatial working memory, and spatial memory. Once again, these relationships were sex-dependant. In the sample as a whole, a high depression score was correlated with a poor sustained attention score. In females, a high depression score was correlated with poorer performances in spatial memory and spatial working memory but was not significantly correlated with sustained attention. In males, a high depression score was correlated with better spatial memory scores. These results echoed the correlation between high anxiety and better sustained attention in males described above and might have similar explanations.

### Cardiovascular function as a potential mediator of the relationships between anxiety-depression and cognition

In the present study, we sought to assess the relationships between cardiovascular function, anxiety, depression, and cognition. We first examined the factors correlated with endothelial function, as a first step towards understanding the various relationships.

#### Endothelial function was correlated with anxiety but not depression

Low endothelial function was correlated with high trait anxiety. In males, high anxiety trait correlated with low endothelial function, whereas in female, high anxiety state but not trait correlated with low endothelial function. Although trait anxiety and state anxiety reflect different dimensions, they are known to be strongly related, as also observed in our present results. Anxiety is related to stress responsiveness and might be the product of the anxiety trait and a stressor [35]. According to our present observations, endothelial function might be correlated with state anxiety because the latter results from the interaction between a personality trait and environmental challenges. Our results suggest that a subclinical relationship between anxiety and cardiovascular function could already present in young adults and expressed differently in males and females.

We did not find a correlation between the depression score and endothelial function. Indeed, in anxiety and depression, autonomic dysfunctions might contribute to an increase in the cardiovascular risk [13, 36, 37]. However, a study of patients with a major depressive disorder found that the concomitant presence of anxiety is associated with greater disturbance of the autonomic nervous system [38]. Thus, autonomic impairments might be triggered more by anxiety than by depression; this might explain why endothelial function was correlated with anxiety but not depression.

#### Endothelial function was not correlated with cognition

The relationship between cognition and cardiovascular function is already apparent in middle-aged adults [39]. However, our results did not indicate that endothelial function is significantly related to cognitive performances in young adults from the general population. The absence of a significant correlation might be due to several reasons: 1) the fact that atherosclerotic lesions take a long time to form [40]; 2) the sample size of our population; 3) a relatively healthy young population.

It will therefore be important to study these relationships at different stages in adulthood and thus determine when the relationships between cognition and cardiovascular function start to emerge. A longitudinal approach would also help us understand whether or not cardiovascular function is involved in anxiety, depression, and the associated cognitive impairments. Indeed, the biological pathways through which emotional disorders impact cognition are complex and subject to much debate [10, 41, 42].

#### Is endothelial function a mechanism by which anxiety and depression lead to cognitive impairments?

Several studies suggested that anxiety and depression lead to pathological cardiovascular dysfunction [11, 12]. Thus, one can hypothesize that endothelial dysfunction is one of the routes through which anxiety and depression affect cognition [10]. To address this question, it is essential to investigating these variables concomitantly. However, the role of endothelial function in these mechanisms has barely been investigated. Our present results did not highlight a link between endothelial function and cognitive modifications in young adults from the general population.

Nevertheless, our exploratory study provided novel findings about young adults. To explore these relationships and cardiovascular mechanisms, more studies are needed in different adult populations. Longitudinal studies would also provide a better understanding of these relationships and enable long-term predictions.

### Sex differences

Major sex differences in the prevalence and manifestation of emotional disorders (including cognitive and autonomic symptoms) have been reported [1, 2]. Cardiovascular diseases affect males and females differently in terms of age of onset, risk factors, and pathophysiological mechanisms [43]. Although sex is a biological variable of major importance, many studies do not include participants with the appropriate sex ratio and do not consider the sex differences evidenced in preclinical or clinical research [44]. This leads to an incomplete understanding of diseases mechanisms and comorbidities and thus to suboptimal diagnoses and treatments.

Our present results support not only sex differences in anxiety and depression scores but also in the associations between anxiety, depression, cognitive performance, and endothelial function. There is also a need to report sex-differentiated data and to take account of the participant’s sex as a biological variable that influences the accuracy and interpretation of study results. The mechanisms may not necessarily be the same in male and female sexes, and sex-specific healthcare and prevention strategies can be considered.

### Study limitations and strengths

The present study has several limitations. Firstly, our sample size was quite small; this doubtless reduced the study’s statistical power (especially in analyses by sex) and prevented us from controlling for many confounding variables. Secondly and importantly, most of our results concerned correlations; even though the literature data suggest that the variables studied here are causally related, our study design prevented us from drawing conclusions about causality.

Our study also had a number of strengths. Firstly, we studied young adults—a population that is rarely considered in this field. Secondly, we used validated psychometric tools to measure levels of anxiety, depression, and cognition. Thirdly, we used a validated, largely operator-independent method to assess endothelial function, which is often difficult to measure accurately with other methods. Fourthly, to our knowledge, the present study is the first to explore the relationships between anxiety, depression, cognition, and endothelial function concomitantly in young adults. If the present results are confirmed and extended, they might be clinically significant for the prevention of comorbidities in adulthood.

## Conclusion

The results of our exploratory study suggest that there are relationships (including sex-specific relationships) between anxiety/depression, cognition, and endothelial function in young adults from the general population. There is growing interest in assessing the dynamic links between these variables; indeed, a better understanding of early-onset relationships might facilitate preventive strategies. Further research is thus needed to deepen our results in healthy young adults and to study the underlying mechanisms over the long term. Furthermore, a sex-specific approach should be adopted in this context.
